# Advanced nanoporous TiO_2_ photocatalysts by hydrogen plasma for efficient solar-light photocatalytic application

**DOI:** 10.1038/srep29683

**Published:** 2016-07-13

**Authors:** Ha-Rim An, So Young Park, Hyeran Kim, Che Yoon Lee, Saehae Choi, Soon Chang Lee, Soonjoo Seo, Edmond Changkyun Park, You-Kwan Oh, Chan-Geun Song, Jonghan Won, Youn Jung Kim, Jouhahn Lee, Hyun Uk Lee, Young-Chul Lee

**Affiliations:** 1 Advanced Nano-surface Research Group, Korea Basic Science Institute, Daejeon 305-806, Republic of Korea; 2 Sustainable Bioresource Research Center, Korea Research Institute of Bioscience and Biotechnology (KRIBB), Daejeon 305-806, Republic of Korea; 3 Department of Applied Chemistry and Biological Engineering, Chungnam National University, Daejeon 305-764, Republic of Korea; 4 Division of Bio-Analytical Science, Korea Basic Science Institute (KBSI), Daejeon 305-806, Republic of Korea; 5 Biomass and Waste Energy Laboratory, Korea Institute of Energy Research (KIER), 152 Gajeong-ro, Yuseong-gu, Daejeon 305-343, Republic of Korea; 6 Central Laboratory, Andong National University, Gyeongsangbuk-do 36729, Republic of Korea; 7 Department of BioNano Technology, Gachon University, Gyeonggi-do 13120, Republic of Korea

## Abstract

We report an effect involving hydrogen (H_2_)-plasma-treated nanoporous TiO_2_(H-TiO_2_) photocatalysts that improve photocatalytic performance under solar-light illumination. H-TiO_2_ photocatalysts were prepared by application of hydrogen plasma of assynthesized TiO_2_(a-TiO_2_) without annealing process. Compared with the a-TiO_2_, the H-TiO_2_ exhibited high anatase/brookite bicrystallinity and a porous structure. Our study demonstrated that H_2_ plasma is a simple strategy to fabricate H-TiO_2_ covering a large surface area that offers many active sites for the extension of the adsorption spectra from ultraviolet (UV) to visible range. Notably, the H-TiO_2_ showed strong **·**OH free-radical generation on the TiO_2_ surface under both UV- and visible-light irradiation with a large responsive surface area, which enhanced photocatalytic efficiency. Under solar-light irradiation, the optimized H-TiO_2_ 120(H_2_-plasma treatment time: 120 min) photocatalysts showed unprecedentedly excellent removal capability for phenol (Ph), reactive black 5(RB 5), rhodamine B (Rho B) and methylene blue (MB) — approximately four-times higher than those of the other photocatalysts (a-TiO_2_ and P25) — resulting in complete purification of the water. Such well-purified water (>90%) can utilize culturing of cervical cancer cells (HeLa), breast cancer cells (MCF-7), and keratinocyte cells (HaCaT) while showing minimal cytotoxicity. Significantly, H-TiO_2_ photocatalysts can be mass-produced and easily processed at room temperature. We believe this novel method can find important environmental and biomedical applications.

Titanium dioxide (TiO_2_) as a semiconductor material utilizes light to drive photocatalytic reactions for practical applications including organic contaminant degradation in air or water^1^,^2^,^3^. TiO_2_ photocatalysts have attracted much attention over many years due to their strong optical absorptivity, chemical stability, low cost and high reactivity^4^,^5^,^6^,^7^,^8^. A bare TiO_2_ photocatalyst, however, is active only under UV light (λ < 380 nm), which corresponds to less than 4% of natural solar-light. For this reason, an enormous amount of research has been devoted to the enhancement of the visible-light absorptivity of TiO_2_^9^,^10^,^11^. For instance, doping of heteroatoms such as transition metals^12^, nitrogen^13^, sulfur^14^ and phosphorus^15^ into TiO_2_ has been reported. In the past, our group has utilized the modified ultrasound irradiation method to fabricate carbon sulfur-doped nanoporous TiO_2_ exhibiting superior visible-light photocatalytic activities^16^.

Recently, TiO_2_ modification by hydrogen also has received attention^17^,^18^,^19^,^20^. Zheng *et al*. reported that hydrogenated TiO_2_ expands the light-absorption spectra and enhances the separation of photoelectrons and holes^21^. Hydrogenated TiO_2_ has been fabricated via various methods such as hydrogen thermal treatment^22^, chemical reduction and oxidation^23^, electrochemical reduction^24^, and anodization-annealing^25^. Also, there have been several reports related to hydrogenated TiO_2_ with porous structures to further improve photocatalytic efficiency of TiO_2_^26^,^27^. Despite the interest in such findings on hydrogen modification and porous structures of TiO_2_, it generally complicates the manufacturing process and the results at high cost. Therefore, the development of facile methods for the preparation of advanced TiO_2_ photocatalysts is still highly desirable.

In this study, we performed the fabrication of hydrogenated nanoporous TiO_2_ (H-TiO_2_) that covers a large surface area (427.5 m^2^/g) using a hydrogen (H_2_) plasma treatment system. The hydrogenation, crystallization and porous structure of TiO_2_ are achievable using the H_2_ plasma system, which can be easily applied to a large area of TiO_2_ without annealing. The hydrogenation can improve optical property so that H-TiO_2_ photocatalyst can be operated from UV to visible light. Also, the porosity of H-TiO_2_ photocatalysts can provide many active sites to extend the adsorption area, leading to superior photocatalytic performance. Consequentially, H-TiO_2_ photocatalysts show higher photocatalytic efficiency by 4 times with respect to the degradation of organic compounds in water than those of other commercial TiO_2_ (P25) and as-synthesized TiO_2_. The water purified by H-TiO_2_ was further evaluated in an *in vitro* cytotoxicity test which measures the level of water purification^28^ and monitors by-products after the photocatalytic treatment. This preliminary study served to highlight the potential of mass-production of nanoporous photocatalysts with a high coverage of surface area for environmental and biomedical applications.

## Results

### Morphological characterization and specific H-TiO_2_-formation mechanism

**Figure 1:**
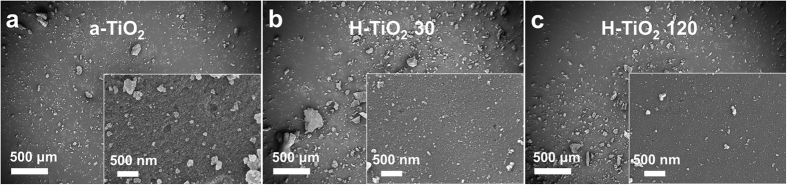
FESEM images of (**a**) as-synthesized TiO_2_ (a-TiO_2_), (**b**) H-TiO_2_ 30, and (**c**) H-TiO_2_ 120.

In this work, we derived a facile strategy for synthesis of hydrogenated TiO_2_ nanoparticles containing a number of pores (see Supplementary Fig. S1). H-TiO_2_ nanoparticles were synthesized from the reaction of hexadecyltrimethylammonium bromide (CTAB) with titanium (IV) butoxide with no additional heat treatment. H-TiO_2_ synthesis entails the following steps: (i) surfactants (CTAB) are dissolved in distilled water to produce micelles acting as nanopore structures in the formation of TiO_2_; (ii) TiO_2_ precursor is added to the surfactant solution in a sol-gel process; (iii) this mixture is treated with H_2_ plasma to remove the micelles and to synthesize crystalline TiO_2_ photocatalysts. Morphological observations of the TiO_2_ samples were conducted using field emission scanning electron microscopy (FESEM). As shown in Fig. 1a–c, the grain sizes were approximately 28 nm for a-TiO_2_ (as-synthesized TiO_2_), 20 nm for H-TiO_2_ 30 (H_2_ plasma treatment time: 30 min), and 18 nm for H-TiO_2_ 120.

**Figure 2:**
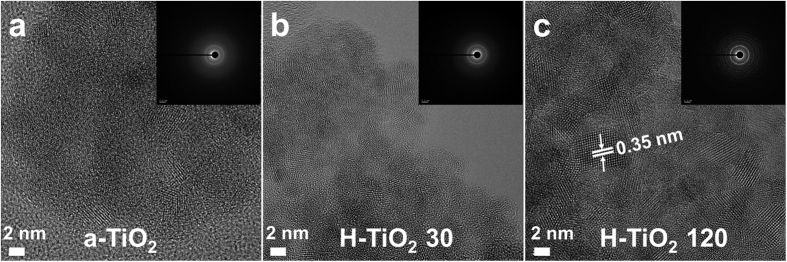
HR-TEM images of (**a**) as-synthesized TiO_2_ (a-TiO_2_), (**b**) H-TiO_2_ 30, and (**c**) H-TiO_2_ 120, with inset showing SAED patterns.

The particle sizes of H-TiO_2_ samples are smaller than those of a-TiO_2_ due to the micelle degradation by H_2_ plasma, which results in the morphological changes of H-TiO_2_ to the irregular structure of aggregated nanoparticles^29^. The formation of the nanoporous structures results from such interconnection of H-TiO_2_ nanoparticles^30^. The high-resolution transmission electron microscopy (HRTEM) image in Fig. 2 confirms the high crystallinity of the TiO_2_ samples. Especially, the apparent lattice fringes clearly indicates the formation of highly anatase/brookite bicrystallized H-TiO_2_ 120. The selected area diffraction (SAD) patterns show that all the samples have the identical lattice spacing (d = 0.35 nm, corresponding to the (101) plane of anatase poly-crystal phase)^31^,^32^ and very similar diffraction patterns. On the basis of the above results, the internal pores were created by surfactant-assisted H_2_ plasma^29^,^30^,^33^. In order to investigate the pore distributions of the TiO_2_ samples, a Brunauer-Emmett-Teller (BET) analyzer was employed to obtain the BET surface areas, which were 36.4 m^2^/g for commercial TiO_2_, 62.3 m^2^/g for a-TiO_2_, 271.8 m^2^/g for H-TiO_2_ 30, and 427.5 m^2^/g for H-TiO_2_ 120 (see Supplementary Table S1). Recently, Ioannidou *et al*. reported hydrogenated commercial TiO_2_ photocatalysts prepared by heat-treatment at 400–800 °C under flowing hydrogen and their BET surface area values were ranging from 2 to 107 m^2^/g^26^. Also, Yuan *et al*. performed the fabrication of hydrogenated TiO_2_ mesoporous spheres by annealing in hydrogen atmosphere at 400 °C, the BET value exhibited ~152 m^2^/g^27^. In fact, BET surface areas and the pore size distributions are strongly dependent on H_2_ plasma treatments. The H-TiO_2_ 120 exhibited the highest surface area providing many active sites to extend the adsorption spectra from UV to the visible range, which contributes to superior photocatalytic activity. This result indicates that H_2_ plasma plays a crucial role in TiO_2_ pore formation and the crystalline phase.

### X-ray diffraction (XRD) patterns and Raman spectra

**Figure 3:**
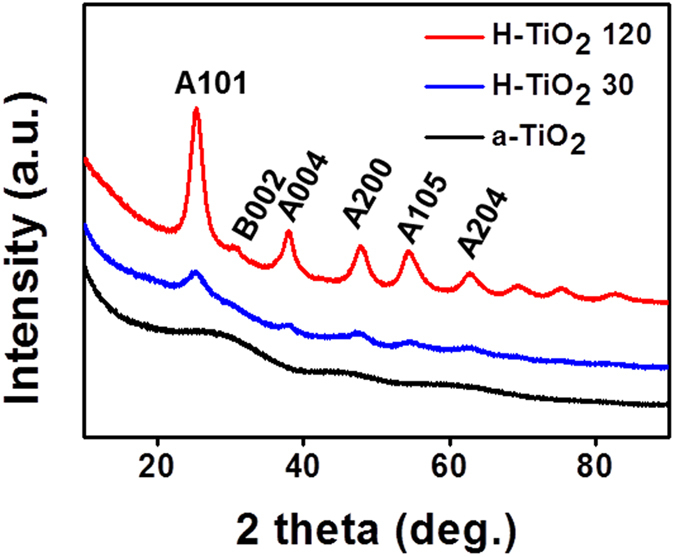
XRD patterns of as-synthesized TiO_2_ (a-TiO_2_), H-TiO_2_ 30, and H-TiO_2_ 120.

X-ray diffraction (XRD) and Raman analysis reconfirmed that the crystal structures of the final stage of the synthesized samples. Figure 3 shows the XRD patterns obtained from a-TiO_2_, H-TiO_2_ 30, and H-TiO_2_ 120. The spectrum of a-TiO_2_ presents weak and broad peaks around 30° and 48° corresponding to the (002) of bookite and the (200) of anatase TiO_2_ phases, respectively. The characteristic peaks of H-TiO_2_ 30 and H-TiO_2_ 120 were observed at 2θ = 25.4°, 38.0°, 47.9°, 54.3°, and 62.8°, corresponding to the (101), (004), (200), (105), and (204) planes of the anatase TiO_2_ phases (space group I4_1_/amd, JCPDS card No. 841286), respectively. Finally, the peak at 30.7° corresponds to the (002) plane of the brookite H-TiO_2_ phases (space group P*cab*, JCPDS card No. 121360)^16^,^29^,^34^. In particular, the crystallinity of the H-TiO_2_ was enhanced with the increasing H_2_ plasma treatment time. The XRD results indicate that all of the H-TiO_2_ samples are composed of anatase/brookite phases, which implies that the H_2_ plasma enhanced the crystallinity of TiO_2_ due to the high-energy reaction of the plasma species on TiO_2_^29^. The same conclusion was drawn from Raman spectroscopy (see Supplementary Fig. S2). According to group factor analysis, anatase has six Raman active modes (A_1g_ + 2B_1g_ + 3E_g_). Ohsaka *et al*. determined the six modes at 144 cm^−1^ (E_g_), 197 cm^−1^ (E_g_), 399 cm^−1^ (B_1g_), 513 cm^−1^ (A_1g_), 519 cm^−1^ (B_1g_), and 639 cm^−1^ (E_g_) from the Raman spectra of an anatase crystal^35^,^36^,^37^. Our Raman results agree with the previous studies, which reveals that H-TiO_2_ 30 ad H-TiO_2_ 120 are anatase and highly crystalline. Thus, the XRD patterns and the Raman spectra results are consistent with the HR-TEM images, indicating that H-TiO_2_ samples are composed of nanoporous structures with a high-crystalline anatase/brookite phase.

### High-resolution-X-ray photoelectron spectroscopy (HR-XPS) surface analysis

**Figure 4:**
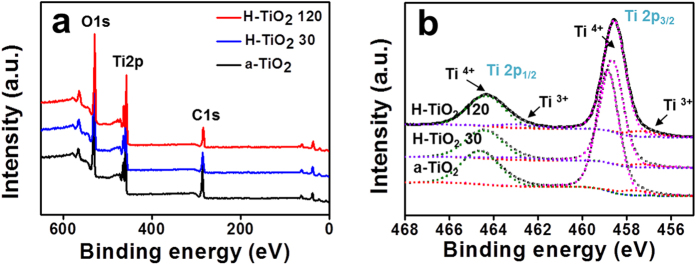
(**a**) Wide-scan and (**b**) Ti 2p HR-XPS spectra of as-synthesized TiO_2_ (a-TiO_2_), H-TiO_2_ 30, and H-TiO_2_ 120.

We next performed X-ray photoelectron spectroscopy (XPS) studies to examine the effect of H_2_ plasma on the chemical states of TiO_2_. Figure 4a shows that C 1s, O 1s and Ti 2p were detected from a-TiO_2_, H-TiO_2_ 30, and H-TiO_2_ 120. The high-resolution Ti 2p XPS spectra of the TiO_2_ samples are plotted in Fig. 4b. Two broad peaks centered at ~464.7 and ~458.8 eV, corresponding to the characteristic Ti 2p_1/2_ and Ti 2p_3/2_ peaks of Ti^4+^ were observed for all of the samples^6^,^8^,^38^. After H_2_ treatment, the Ti 2p peaks of the H-TiO_2_ lead to a negative shift toward the lower binding energies, suggesting that oxygen vacancies (Ti^3+^ sites) are created in H-TiO_2_ during hydrogenation^21^,^39^,^40^. Zheng *et al*. reported that the lower-energy peak of H-TiO_2_ is attributed to the surface Ti–H bonds formed under hydrogen atmosphere. This implies that the release of H_2_ creates a different bonding environment of TiO_2_ such as partial reduction of TiO_2_ under reduced conditions^21^. Taking into account the chemical compositions of the TiO_2_ samples (see Supplementary Table S2), it can be seen that the atomic concentrations of C 1s were decreased as the H_2_ plasma treatment time increased. This indicates that the H_2_ plasma causes the degradation of CTAB containing a large amount of carbon. To understand this phenomenon better, we conducted energy-dispersive X-ray (EDX) mapping of the O, Ti, and C elemental analysis for the a-TiO_2_ and H-TiO_2_ 120 samples (see Supplementary Fig. S3). Apparently, O and Ti have a uniform distribution over the entire TiO_2_ aggregates. Also, the quantity of carbon is decreased in H-TiO_2_ due to CTAB. CTAB micelles are released during H_2_ plasma treatment as illustrated in Fig. S1, which leads to numerous nanopores in the TiO_2_ and a corresponding nanoporous structure^29^,^30^,^33^.

### Optical properties of H-TiO_2_ photocatalysts and photocatalytic mechanism

**Figure 5:**
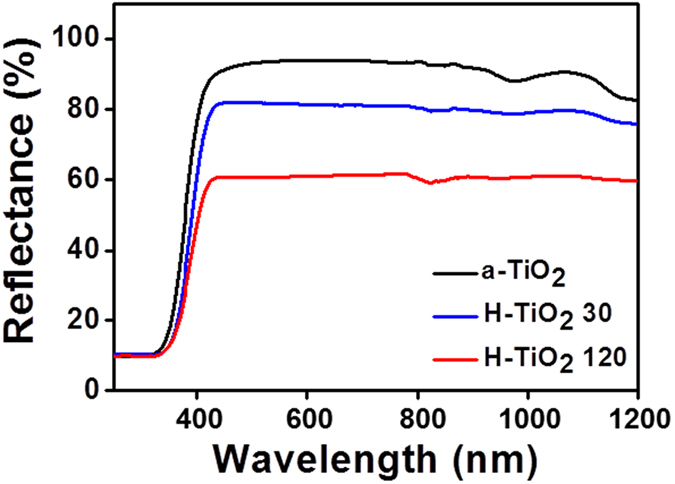
UV-Vis-NIR reflectance of as-synthesized TiO_2_ (a-TiO_2_), H-TiO_2_ 30, and H-TiO_2_ 120.

**Figure 6:**
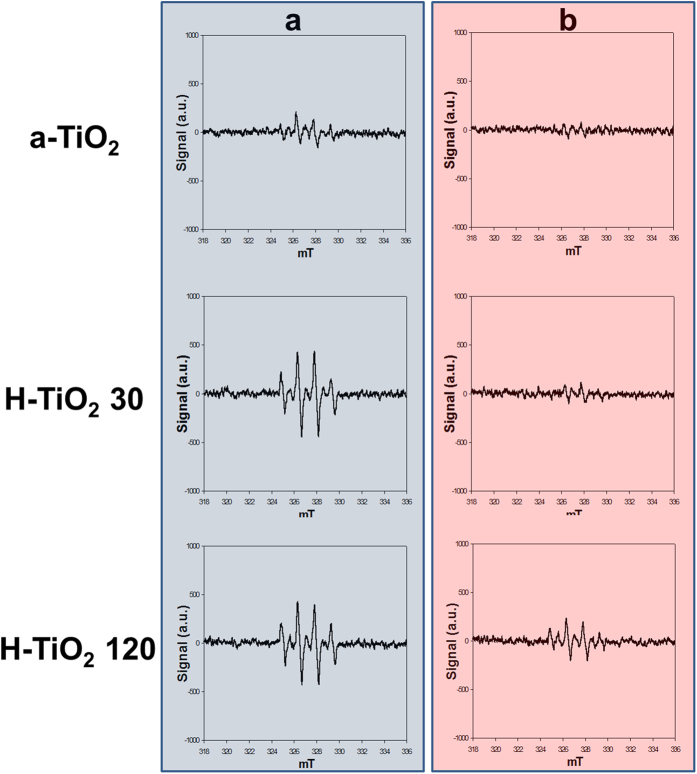
ESR spectra of as-synthesized TiO_2_ (a-TiO_2_), H-TiO_2_ 30, and H-TiO_2_ 120 at (**a**) 365 nm and (**b**) LED irradiation.

The photocatalytic efficiency of normal TiO_2_ is limited by its wide band gap and the low efficiency of the recombined electrons and holes^1^,^2^,^21^. In the present study, we purposed to increase visible-light absorption using H_2_ plasma treatment to narrow the band gap or to form localized states therein (see Supplementary Fig. S4). The formation of Ti-H and Ti-OH bonds on the surface of hydrogenated TiO_2_ nanoparticles can improve the separation of electrons and holes^41^. In an investigation of the optical properties of the a-TiO_2_, H-TiO_2_ 30, and H-TiO_2_ 120 photocatalysts, the ultraviolet-visible-near infrared (UV-Vis-NIR) reflectance (%) between 250 nm and 1200 nm was measured as shown in Fig. 5. The absorbance spectra for all of the TiO_2_ samples exhibited UV-light absorption below the 400 nm in wavelength^42^. The increasing of oxygen vacancies or Ti^3+^ species results in a narrowing bandgap during H_2_ plasma treatment and increases the visible light absorption of the H-TiO_2_^16^,^43^,^44^. To further investigate the improved photocatalytic capability of H-TiO_2_, the electron spin resonance (ESR) technique was employed to detect O_2_^−**·**^ and **·**OH free-radical generation. These radicals can attack organic substrates, leading to their degradation in water^45^,^46^. Figure 6 plots the ESR spectra of the a-TiO_2_, H-TiO_2_ 30 and H-TiO_2_ 120 photocatalysts under UV light (365 nm wavelength) and visible-light (i.e., LED) irradiation. When the UV light was irradiated for 5 min, all the TiO_2_ photocatalysts displayed 1:2:2:1 patterns indicating the production of **·**OH free radicals^47^. In the case of the LED irradiation, only weak and negligible **·**OH free-radical peaks were observed in a-TiO_2_. However, the peak intensity of the H-TiO_2_ photocatalyst was increased with increasing H_2_ plasma treatment time. This was due to the presence of the active oxygen species. The oxygen vacancy states exist within the band gap of H-TiO_2_ photocatalyst and these electronic states as the intermediate facilitate the two-step excitation from the valence band to the conduction band under the visible light^16^,^47^,^48^,^49^.

### Photocatalytic degradation of azo dye

**Figure 7:**
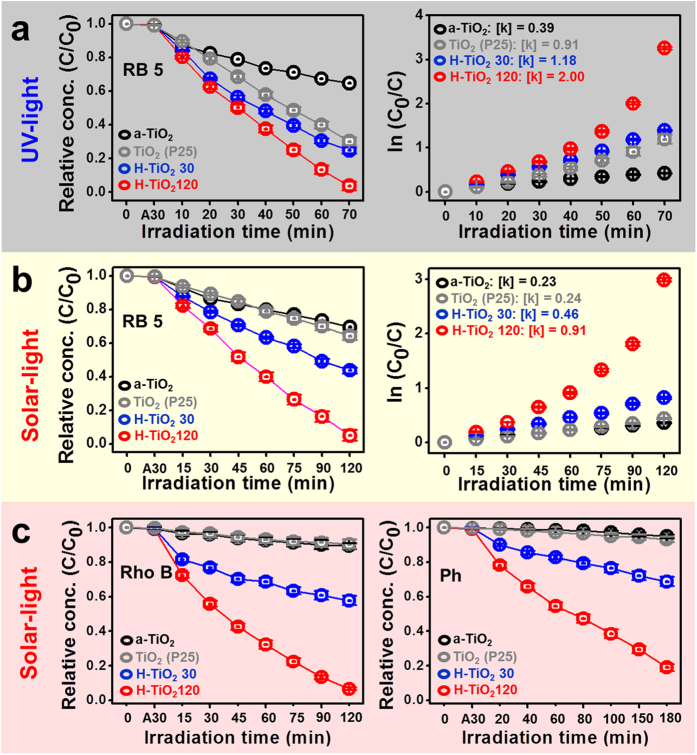
Removal of RB 5, Rhodamine B (Rho B), and Phenol (Ph) by commercial TiO_2_, as-synthesized TiO_2_ (a-TiO_2_), H-TiO_2_ 30, and H-TiO_2_ 120 under UV- and/or solar-light irradiation.

Organic pollutants emitted from various sources give rise to serious ecological problems because the degradation of these pollutants is often slow and traditional removal treatments are usually ineffective and not environmentally interconvertible^46^. To examine the effects of the photocatalytic performance of the a-TiO_2_ and H-TiO_2_ photocatalysts as the attractive means to solve these problems, a degradation test of Ph, Rho B, RB 5 and MB solutions was carried out under UV- and/or solar-light irradiation. The basic photocatalytic mechanism for the degradation of organic pollutants is as the following. The charge separation happens due to excitation of the valence band electrons to the conduction band by the input of ultra-band gap energy. The separate charges then migrate to the surface of the TiO_2_, participating in the redox reactions. The oxygen molecule obtains the electron from the conduction band, forming O_2_^−**·**^ free radicals. The strong reactive oxygen species such as O_2_^−**·**^ and **·**OH can attack pollutant species, leading to their degradation^46^. As shown in Fig. 7a, after 70 min of solar-light irradiation, not only did H-TiO_2_ 30 exhibit good degradation efficiency, but also the H-TiO_2_ 120 photocatalysts almost completely removed the RB 5 (>99% efficiency). The degradation rate, *k*, related to the degradation efficiency, was 0.39 h^−1^ for a-TiO_2_, 0.91 h^−1^ for commercial TiO_2_ (P25), 1.18 h^−1^ for H-TiO_2_ 30, and 2 h^−1^ for H-TiO_2_ 120. Similarly, the H-TiO_2_ 120 photocatalysts showed almost complete degradation of the RB 5 solutions under 70 min solar-light irradiation (Fig. 7b), while the other photocatalysts showed relatively low degradation efficiencies. The degradation rates were 0.23 h^−1^ for a-TiO_2_, 0.24 h^−1^ for commercial TiO_2_ (P25), 0.46 h^−1^ for H-TiO_2_ 30, and 0.91 h^−1^ for H-TiO_2_ 120.

Also, extra degradation tests of Rho B and Ph under solar-light irradiation displayed the analogical results. As shown in Fig. 7c, the contaminated Rho B and Ph solutions were almost completely purified by H-TiO_2_ 120 after 120–180 min solar-light irradiation. Such superior photocatalytic performance of H-TiO_2_ can be attributed to its narrowed bandgap, which is supported by formation of many **·**OH free radicals and large surface area of H-TiO_2_^16^,^29^,^49^,^50^. These suggested that H-TiO_2_ could produce many active sites for adsorption of azo dyes on surface of H-TiO_2_, which contributes to the improvement of photocatalytic performance.

The initial duration of solar-light irradiation was 70 min and at the end of each cycle, H-TiO_2_ 120 decolorization was measured (Fig. S5). After 10 repeatable measurements under solar-light irradiation, the photocatalytic conversion ratio of H-TiO_2_ 120 for RB 5 remained approximately 92%. The slight decrease of the conversion ratio after each cycle can be attributed to the loss of the H-TiO_2_ 120 photocatalyst. It is certain that H-TiO_2_ 120 is an outstanding photocatalyst since the degradation efficiency remained constant after the repeated cycles.

### Biocompatibility of H-TiO_2_ photocatalysts

We further conducted an *in vitro* cytotoxicity test to monitor by-products in purified water and to measure the safety level, which is relevant to the reuse of ventilated water. Here, MB-treated water samples were used. Preparatorily, the elimination efficiency of MB was investigated as shown in Fig. S6. H-TiO_2_ 120 exhibited the highest degradation rate (0.61 h^−1^) 150 min after solar-light illumination among the photocatalysts (others: 0.09 h^−1^ for a-TiO_2_, 0.12 h^−1^ for commercial TiO_2_, and 0.30 h^−1^ for H-TiO_2_ 30), which notably showed almost perfect MB degradation. As described above, the structural properties and the excellent solar-light activities of nanoporous H-TiO_2_ 120 photocatalyst allowed us to enhance the photocatalytic performance for MB degradation^16^,^29^,^49^,^50^.

**Figure 8:**
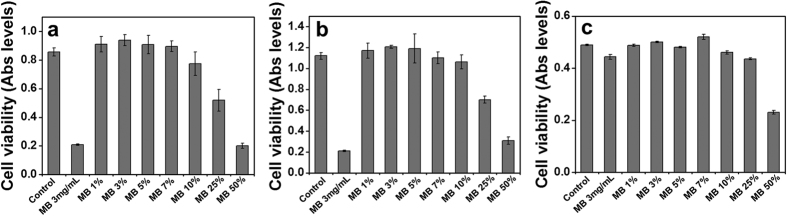
Cytotoxicity of purified water by H-TiO_2_ 120 photocatalysts as analyzed by MMT assay using (**a**) HeLa (cervical cancer cells, human), (**b**) MCF-7 (breast cancer cells, human), and (**c**) HaCaT (keratinocyte cells, human) cell lines.

The waters purified by H-TiO_2_ 120, the performances of which ranged from 0 (MB 3 mg/mL) to 100%, were collected for evaluation of their safety for human cells; specifically, their cytotoxicities were examined by 3-[4,5-dimethylthiazol-2-yl]-2,5 diphenyl tetrazolium bromide (MMT) assay (Fig. 8). Three different cells including HeLa (immortal cell line, human), MCF-7 (breast adenocarcinoma cell line, human), and HaCaT (keratinocyte cell line, human) cells were incubated with the treated water solutions for 24 h. When the purification degree was lower than 90% (MB 10%), the cell viabilities were gradually reduced to zero by MB toxicity or by intermediate by-products harmful to organs in the water. At the purification degree of 90%, the cell viability remained high: over 86% for HeLa cells, 92% for MCF-7 cells, and 90% for HaCaT cells. We found that the water purified (to degrees up to 90%) by the H-TiO_2_ 120 photocatalyst left non- or minimal cytotoxicity in the cells^51^. This result confirms that the water purified by the H-TiO_2_ 120 photocatalysts is safe for humans.

## Discussion

We prepared mass-producible hydrogenated nanoporous TiO_2_ photocatalysts (H-TiO_2_) using H_2_ plasma treatment system without thermal processing. The primary role of H_2_ plasma is to provide TiO_2_ photocatalysts with high crystallinity and many pores for large surface area, thereby generating a great deal of oxygen species for photocatalytic effects. The structural and morphological analysis of the H-TiO_2_ suggest that H_2_ plasma serve the high-bicrystalline phase (anatase/brookite) and a lot of pores for TiO_2_. Especially, under optical examination, the plasma-treated H-TiO_2_ for 120 min (H-TiO_2_ 120) displayed the higher visible-adsorption spectra and the strongest **·**OH free-radical peaks among the photocatalysts, which indicates that H-TiO_2_ 120 has a greater photocatalytic potential in the visible-light regions than commercial TiO_2_ (P25), as-synthesized TiO_2_ (a-TiO_2_) or H-TiO_2_ 30. The H-TiO_2_ 120 photocatalysts, correspondingly, exhibit higher degradation efficiencies for Ph, Rho B, RB 5 and MB solutions and the water purified (to degrees up to 90%) by H-TiO_2_ 120 provides a safe, minimal-cytotoxicity environment for growth of cervical cancer cells (HeLa), breast cancer cells (MCF-7), and keratinocyte cells (HaCaT). Our results showed that H_2_ plasma treatment can be considered as a facile hydrogenation method to produce modified TiO_2_ photocatalysts at room temperature and the H-TiO_2_ photocatalyst has interesting photophysical properties involving high crystallinity and porous structure as it enables photocatalytic purification of organics from water, including those operating with visible light.

## Methods

### Fabrication of H-TiO_2_ photocatalysts

All the reagents for synthesis of H-TiO_2_ photocatalysts were used without further purification. First, in order to fabricate TiO_2_ nanoparticles using a sol-gel method, 5 mol titanium (IV) butoxide (Ti (OC (CH_3_)_3_)_4_, Sigma-Aldrich, USA) was dissolved in an aqueous solution of 0.5 mol hexadecyltrimethylammonium bromide (CTAB, C_16_H_33_N (CH_3_)_3_Br, Sigma-Aldrich, USA)^16^. After stirring for 30 min and aging for 24 h, the cloudy solution was washed several times with deionized (DI) water and dried at room temperature for 48 h. To H_2_-plasma treat and to dry TiO_2_ nanoparticles (10 g, as-synthesized TiO_2_: a-TiO_2_), a plasma treatment system (Covance-MP; Femto-Science Co., Korea) consisting of a 13.56 MHz radio-frequency (RF) generator (up to 300 W), electrode, dielectric materials, ceramic substrate, diffuser, sample stage (size: 150 × 150 mm), gas inlet/outlet, and a vacuum system was used. Argon (purity 99.9%; flow rate, 50 sccm) and H_2_ (purity 99.9%; flow rate, 50 sccm) were employed as a carrier gas and a reactive gas, respectively. The H_2_ plasma treatment time was controlled within the 0–120 min range (plasma power: 120 W). We named the H_2_ plasma treated TiO_2_ for 30 min and 120 min as H-TiO_2_ 30 and H-TiO_2_ 120, respectively.

### Characterization of H-TiO_2_ hybrid photocatalysts

The crystalline structures of the H-TiO_2_ samples were investigated by XRD (Rigaku RDA-cA X-ray diffractometer, Japan) using Cu Kα radiation with a nickel filter. The morphology and size distribution of the H-TiO_2_ samples were recorded by FE-SEM (Hitachi; S-4700, Japan) and HR-TEM (JEOL JEM 2200, Japan). Before the analyses, the samples were placed on the surfaces of copper grids and dried under ambient conditions. Raman spectroscopy (Renishaw RM1000-Invia, UK) was performed in a backscattering configuration excited with a visible laser light (wavelength = 514 nm), a notch filter cut-off frequency of 50 cm^−1^, and a focus-spot size of 5 μm. The spectra were collected through a ×100 objective lens and recorded on an 1800 lines per mm^−1^ grating providing a spectral resolution of ≈1 cm^−1^. To avoid laser-induced heating and ablation of the samples, all of the spectra were recorded at low power levels (≈0.1 mW) and over short integration times (≈5 s). The BET surface areas, pore volumes, and pore diameters of the H-TiO_2_ samples were determined using a BET analyzer (Micromeritics ASAP 2020, USA) to investigate specific surface area and the pore size distribution. HR-XPS with monochromatic Al Kα X-ray radiation (hν = 1486.6 eV) operated at 120 W (Kratos Analytical, AXIS Nova, Manchester, UK) was used to investigate the surface properties of the samples. The shift of binding energy resulting from relative surface charging was corrected using the C 1s level at 284.6 eV as an internal standard. Diffuse reflectance measurements were performed using a Shimadzu Lambda 900 spectrophotometer equipped with an integrating sphere. The reflectance spectra were recorded at 190–1200 nm in wavelength. For free-radical detection by 5,5-dimethyl-1-pyrroline N-oxide (DMPO; 0.3 M in PBS buffer at pH 7.2, Sigma-Aldrich, USA) as a spin trap agent, an aliquot of as-prepared sample (100 μL of 5 mg H-TiO_2_ sample mixed with 300 μL DMPO solution) was filled into a capillary tube and directly irradiated with a UV (λ = 365 nm) or light-emitting diode (LED) light (>400 nm) source for 5 min and the results were recorded by ESR spectrometry (JEOL JES-FA200, Japan; center field: 327 mT; power: 1 mW; amplitude: 5.0 × 100; modulation width: 0.4 × 1; sweep width: 1 × 10; sweep time: 30 s).

### Measurement of photocatalytic activities

The photocatalytic degradation of phenol (Ph; 1.88 mg/L, Aldrich, USA), rhodamine B (Rho B; 3 mg/L, Sigma-Aldrich, USA), reactive black 5 (RB 5; 3 mg/L, Sigma-Aldrich, USA) and methylene blue (MB; 3 mg/L, Aldrich, USA) solutions containing H-TiO_2_ samples (0.5 g/L) were carried out under UV- (4 W, 365 nm, VSLAB VL-4CL, Korea) and/or solar-light (150 W Xe lamp, 200 nm > λ, SCHOTT, USA) irradiation. Before the insertion of H-TiO_2_, the solution was stirred for 30 min under illumination (A30). The absorbance of the solutions were measured by UV-VIS-IR spectrometry (Varian, Cary5000, Australia) in the 200–800 nm region^16^. The concentrations of the Ph, Rho B, RB 5 and MB solutions after photo-irradiation were measured from the peak intensities of the absorbance at 270, 555, 598 and 664 nm, respectively^16^. The change in the concentration (ln (C_0_/C) = *k*t, where *k* is the apparent reaction rate constant, and C_0_ and C are the initial and reaction concentrations of RB 5 or MB) of the dye solution with reaction time (0–180 min) was also investigated. To demonstrate the stability of the photocatalysts, H-TiO_2_ samples were recycled. A recycling test of the photocatalytic activity of the H-TiO_2_ samples was performed after washing with DI water and drying in an oven (60 °C) for 6 h after each cycle.

### *In vitro* cytotoxicity test of purified water using H-TiO_2_

The cytotoxicity of the samples was evaluated by MTT assay. Briefly, HeLa (immortal cell line, human), MCF-7 (breast adenocarcinoma cell line, human), and HaCaT (keratinocyte cell line, human) cells were seeded in a 96-well plate at a density of 8 × 10^3^ cells per well and cultured in a humidified incubator at 37 °C for 24 and 72 h under a 5% CO_2_ atmosphere in Dulbecco’s modified Eagle’s medium (DMEM) and/or Roswell Park Memorial Institute (RPMI)-1640 supplemented with 10% FBS and 1% penicillin antibiotics. The DMEM and/or RPMI-1640 media were used to purify water samples (to 0, 50, 75, 90, 93, 95, 97, 99 and 100% degrees of methylene blue (MB) degradation) using the H-TiO_2_ photocatalyst after they were incubated for 24 h. Then, 20 μL of 0.2 mg/mL MTT solution in medium was added to each well and incubated at 37 °C for 2 h. Finally, the optical density (OD) was measured at 490 nm with an absorbance microplate reader (EMax microplate reader, Bucher Biotec AG, Basel, Switzerland).

Preparatory to photocatalytic and cytotoxicity tests, the average of the data was taken after the repeated measurements of four cycles of tests with the mean ± standard deviation. A statistical analysis was performed by analysis of variance (ANOVA), with p-values < 0.05 considered as significant.

## Additional Information

**How to cite this article**: An, H.-R. *et al*. Advanced nanoporous TiO_2_ photocatalysts by hydrogen plasma for efficient solar-light photocatalytic application. *Sci. Rep.* **6**, 29683; doi: 10.1038/srep29683 (2016).

## Supplementary Material

Supplementary Information
