# Supplementary material for: Advanced nanoporous TiO2 photocatalysts by hydrogen plasma for efficient solar-light photocatalytic application
Source: Sci Rep. 2016 Jul 13;6:29683. doi: 10.1038/srep29683 (PMC4942796; doi:10.1038/srep29683)
Supplement: Supplementary Information [file srep29683-s1.pdf]

Supplementary Information

Advanced nanoporous TiO_2 photocatalysts by hydrogen plasma for efficient solar-light photocatalytic application

Ha-Rim An[1,†], So Young Park[1,†], Hyeran Kim[1,†], Che Yoon Lee[1], Saehae Choi[2], Soon Chang Lee[3], Soonjoo Seo[1], Edmond Changkyun Park[4], You-Kwan Oh[5], Chan-Geun Song[1], Jonghan Won[1], Youn Jung Kim[6], Jouhahn Lee[1], Hyun Uk Lee[1,*], Young-Chul Lee[7,*]

[1]Advanced Nano-surface Research Group, Korea Basic Science Institute, Daejeon 305-806, Republic of Korea

[2]Sustainable Bioresource Research Center, Korea Research Institute of Bioscience and Biotechnology (KRIBB), Daejeon 305-806, Republic of Korea

[3]Department of Applied Chemistry and Biological Engineering, Chungnam National University, Daejeon 305-764, Republic of Korea

[4]Division of Bio-Analytical Science, Korea Basic Science Institute (KBSI), Daejeon 305-806, Republic of Korea

[5]Biomass and Waste Energy Laboratory, Korea Institute of Energy Research (KIER), 152 Gajeong-ro, Yuseong-gu, Daejeon 305-343, Republic of Korea

[6]Central Laboratory, Andong National University, Gyeongsangbukdo 760-749, Republic of Korea

[7]Department of BioNano Technology, Gachon University, Gyeonggi-do 13120, Republic of Korea

[†]These authors contributed equally to this work.

Correspondence should be addressed to L.H.U. and L.Y.C. (email: leeho@kbsi.re.kr (L.H.U.), dreamdbs@gachon.ac.kr (L.Y.C.))

Table S1. Hydrogen effects on BET, pore volume and size of commercial TiO_2, as-synthesized TiO_2 (a-TiO_2), H-TiO_2 30 and H-TiO_2 120.

Atomic %	TiO_2 (P25)	a-TiO_2	H-TiO_2 30	H-TiO_2 120
BET (m^2/g)	36.4	62.3	271.8	427.5
Total pore volume (cm^3/g)	0.014	0.019	0.139	0.201
Mean pore diameter (nm)	4.31	3.91	2.05	1.88

Table S2. Chemical compositions of as-synthesized TiO_2 (a-TiO_2), H-TiO_2 30, and H-TiO_2 120.

Atomic %	a-TiO_2	H-TiO_2 30	H-TiO_2 120
C	54.7	48.9	40.0
O	34.6	36.1	43.6
Ti	10.7	15.0	16.4

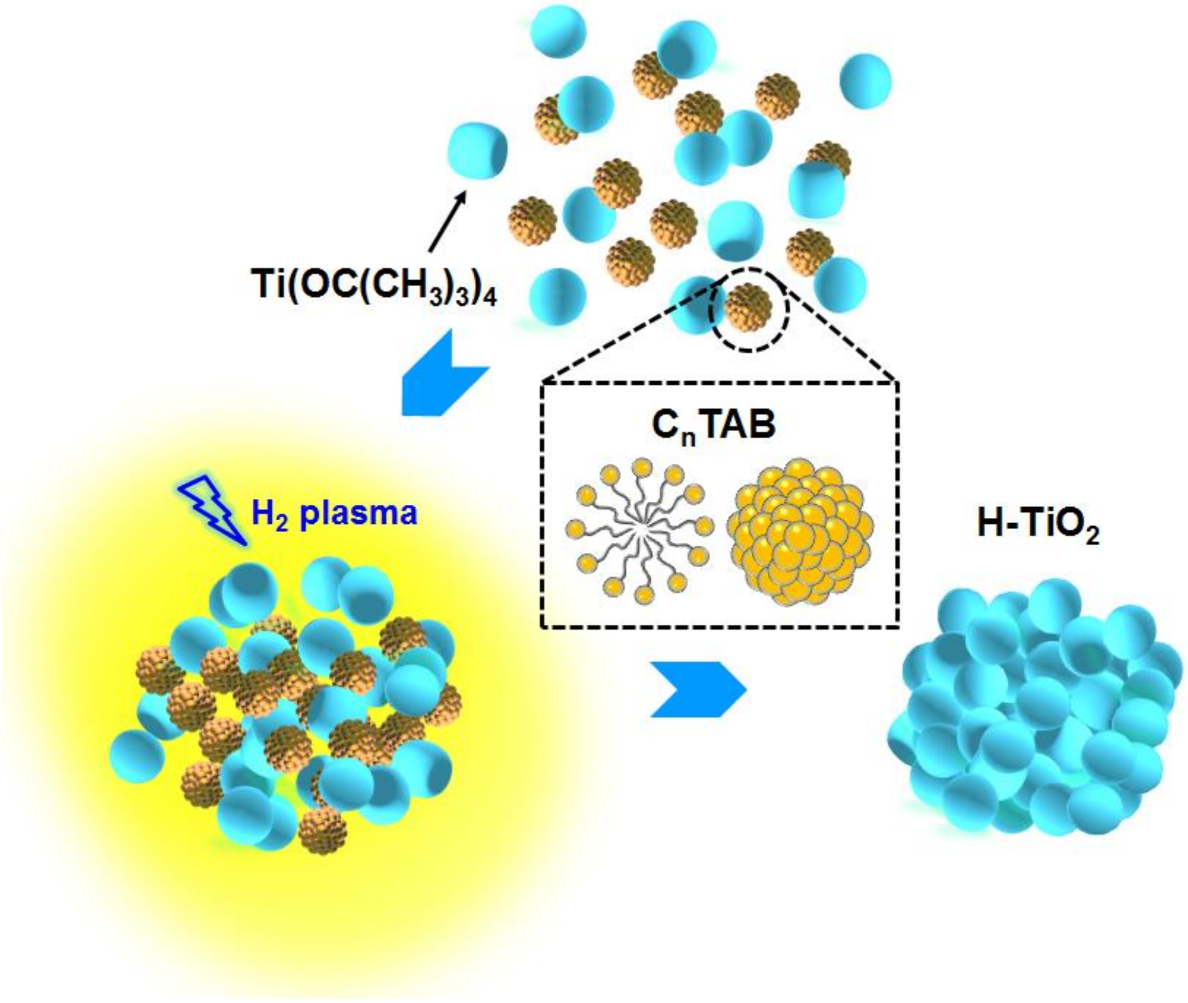

Figure S1. Schematic diagrams of formation mechanism of H-TiO_2 photocatalysts.

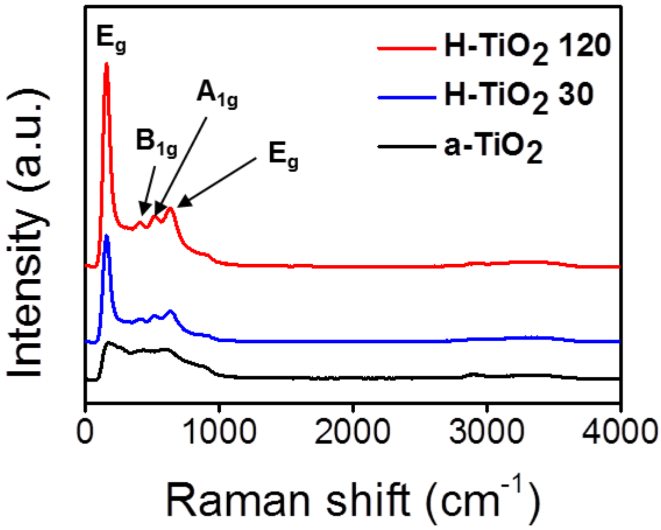

Figure S2. Raman spectra of as-synthesized TiO_2 (a-TiO_2), H-TiO_2 30, and H-TiO_2 120.

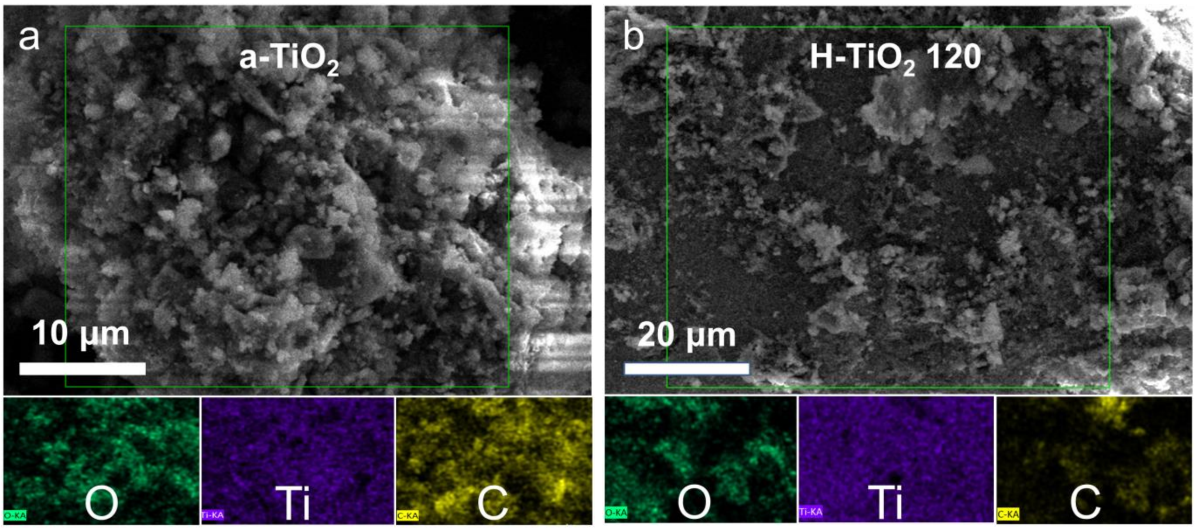

Figure S3. EDX mapping of O (green), Ti (violet), and C (yellow) of (a) as-synthesized TiO_2 (a-TiO_2) and (b) H-TiO_2 120.

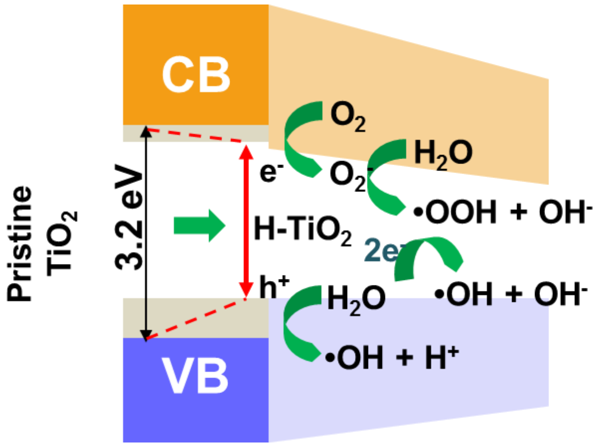

Figure S4. Suggested schematics of photocatalytic mechanism of H-TiO_2 photocatalysts under visible-light irradiation.

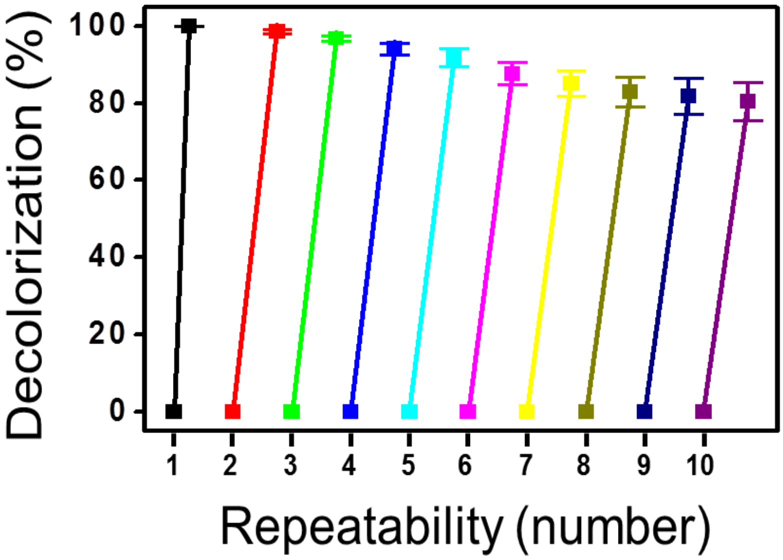

Figure S5. Recycling results for decolorization of RB 5 solution on H-TiO_2 120 photocatalysts.

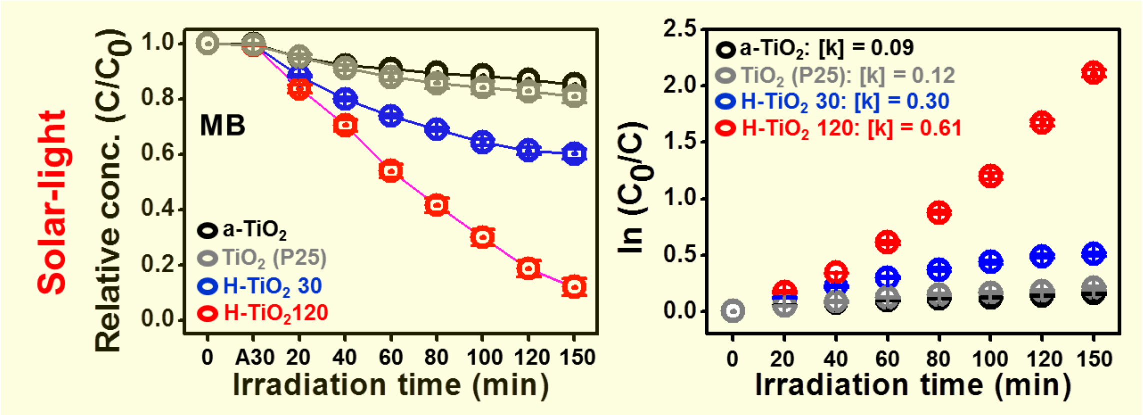

Figure S6. Plots of MB removal by commercial TiO_2, as-synthesized TiO_2 (a-TiO_2), H-TiO_2 30, and H-TiO_2 120 under solar-light irradiation.
